# Designing synthetic microbial communities for enhanced anaerobic waste treatment

**DOI:** 10.1128/aem.00404-25

**Published:** 2025-05-16

**Authors:** Lisa Jourdain, Wenyu Gu

**Affiliations:** 1 MICROBE laboratory, Institute of Environmental Engineering, School of Architecture, Civil and Environmental Engineering, Swiss Federal Institute of Technology 400003 , Lausanne, Switzerland; University of Delaware, Lewes, Delaware, USA

**Keywords:** anaerobic digestion, synthetic community, resource recovery, methanogenesis, syntrophy, fermentation, microbial interaction

## Abstract

Synthetic microbial communities (SynComs) are powerful tools for investigating microbial interactions and community assembly by focusing on minimal yet functionally representative members. Here, we will highlight key principles for designing SynComs, specifically emphasizing the anaerobic digestion (AD) microbiome for waste treatment and upcycling. The AD process has traditionally been used to reduce organic waste volume while producing biogas as a renewable energy source. Its microbiome features well-defined trophic layers and metabolic groups. There has been growing interest in repurposing the AD process to produce value-added products and chemical precursors, contributing to sustainable waste management and the goals of a circular economy. Optimizing the AD process requires a better understanding of microbial interactions and the influence of both biotic and abiotic parameters, where SynComs offer great promise. Focusing on AD microbiomes, we review the principles of SynComs’ design, including keystone taxa and function, cross-feeding interactions, and metabolic redundancy, as well as how modeling approaches could guide SynComs design. Furthermore, we address practical considerations for working with AD SynComs and examine constructed SynComs designed for anaerobic waste digestion. Finally, we discuss the challenges associated with designing and applying SynComs to enhance our understanding of the AD process. This review aims to explore the use of synthetic communities in studying anaerobic digestion and highlights their potential for developing innovative biotechnological processes.

## SynComs AND THEIR APPLICATIONS

Synthetic communities (SynComs) are controlled assemblages of selected microorganisms designed to simplify and replicate the behaviors of natural microbial ecosystems at a specific scale. Initially based on the concept of core microbiome, SynComs aim to create a minimal yet functionally representative consortium that preserves the key ecological and functional roles of the original community (1, 2). Compared to undefined consortia, SynComs offer advantages for mechanistic studies and investigating causal interactions. By reducing the inherent complexity of natural microbiomes, they provide a platform to systematically explore the combinatorial effects of biotic factors and abiotic factors (3) under controlled conditions (4, 5). Through species drop-in and drop-out experiments, SynComs enable the investigation of the roles of individual taxa, interspecies interactions of competition or cross-feeding, and the effects of microbial diversity and redundancy on community stability and resilience (6). Furthermore, the reproducibility of synthetic communities across time and laboratories ensures consistent manipulation of identical communities, advancing experimental rigor and enabling collaborative research. These insights can inform interventions to restore stable microbial communities and address challenges in manipulating functionality in microbial systems (7–9). They also hold promise for developing microbial processes for industrial purposes, including improving biofuel production (10), synthesizing value-added products (11), and developing therapeutic strategies such as probiotics (12).

The design of SynComs does not necessarily require complete mimicry of natural microbiomes but rather focuses on achieving a specific function of interest, tailored to the objectives of the study. Developing such synthetic communities remains challenging due to the rapidly expanding design space that arises as organism diversity and the number of biotic and abiotic factors (e.g., pH, hydraulic retention time, feedstock nature, organic loading rate, inhibitory metabolite accumulation) considered increase. The intricate interplay between community members and environmental factors together shapes a dynamic relationship between community composition and function (5). Furthermore, the metabolic flexibility of microbes, influenced by cultivation conditions and the exploitation of distinct ecological niches, further complicates SynComs design, often resulting in multiple local optima (4, 6).

In this article, we aim to explore the design and applications of SynComs in waste anaerobic digestion (AD) and examine their potential for advancing waste management and biotechnological innovation. We first examine the extent to which synthetic communities can capture and replicate the complex dynamics observed in AD communities by reviewing metabolic functions within AD communities. Next, we explore the emerging applications of SynComs in mechanistic studies, highlighting their developing role in practical contexts by reviewing their design principles. Finally, we summarize available studies to discuss the future potential of SynComs in biotechnological advancements, particularly in waste upcycling and the design of novel processes for anaerobic digestion and related systems. Overall, we aim to highlight SynComs as valuable tools for driving innovation in AD processes.

## ANAEROBIC DIGESTION PROCESS

### As a sustainable technology

The anaerobic food chain refers to the sequential degradation and utilization of biopolymers by a community of microorganisms under oxygen-free conditions. Anaerobic digestion can be driven by different nutrient cycles, and here we focus on a carbon-centered AD process, where methane (CH_4_) is a main final product. These carbon-based trophic networks utilize compounds such as acetate, formate, and hydrogen (H_2_) as terminal electron acceptors and are prevalent in diverse anoxic environments, including the mammalian intestinal tract, stratified estuaries, and anoxic soils and sediments (13). They play a critical role in global carbon cycling, facilitating the transformation of organic matter. In the case of the mammalian digestive system, host health is dependent on the functioning of these food webs, where dysbiosis can lead to nutrient imbalances and the emergence of disease states (14).

This process finds crucial applications in biotechnology for treating organic wastes, such as municipal and industrial wastewater sludge, food waste, and manure. AD not only reduces waste volumes but also generates biogas, primarily CH_4_, as a renewable energy source (15). More recently, the field has evolved to emphasize waste valorization, aiming at producing value-added products from contaminated feedstocks. A key area of interest is repurposing AD to produce short- or medium-chain carboxylic acids (SCCAs or MCCAs) (16, 17). These platform molecules serve as precursors for a wide range of industrial compounds, including esters, ketones, aldehydes, alcohols, and alkanes (14). The SCCA mixture generated from AD can provide carbon substrates for additional microbial processes, leading to the production of biofuels and biopolymers, such as microbial lipids and polyhydroxyalkanoates (PHAs), without the need to separate SCCA species (18). Such processes offer sustainable alternatives to petroleum-based chemicals and plant-derived carbohydrates for the manufacturing of oleochemical and biodegradable plastics (19). SCCA production can be achieved by a shortened digestion process sometimes referred to as anaerobic fermentation (14), where the activity of methanogens is suppressed with a shorter hydraulic retention time or non-neutral pH conditions (15). MCCAs are easier to extract and have greater industrial value (20). Their formation occurs through an elongation process of the carboxylic acid chain, primarily driven by the reverse β-oxidation of acetic acid, n-butyric acid, and caproic acid, using electron donors such as ethanol, lactic acid, methanol, and H_2_. The MCCAs produced through this process mainly include caproic acid, heptylic acid, and caprylic acid (21, 22). Consequently, AD plays a pivotal role in waste management innovation and in advancing circular economy principles by transforming waste streams into valuable resources.

Waste streams contain a diverse array of microorganisms, presenting significant challenges in manipulating and optimizing the AD process to stably and continuously produce targeted compounds. Therefore, there is a growing need for quantitative predictions and precise fine-tuning of AD processes, along with tools that enable better control over product yields and quality.

### Core metabolic groups and interactions

**Fig 1:**
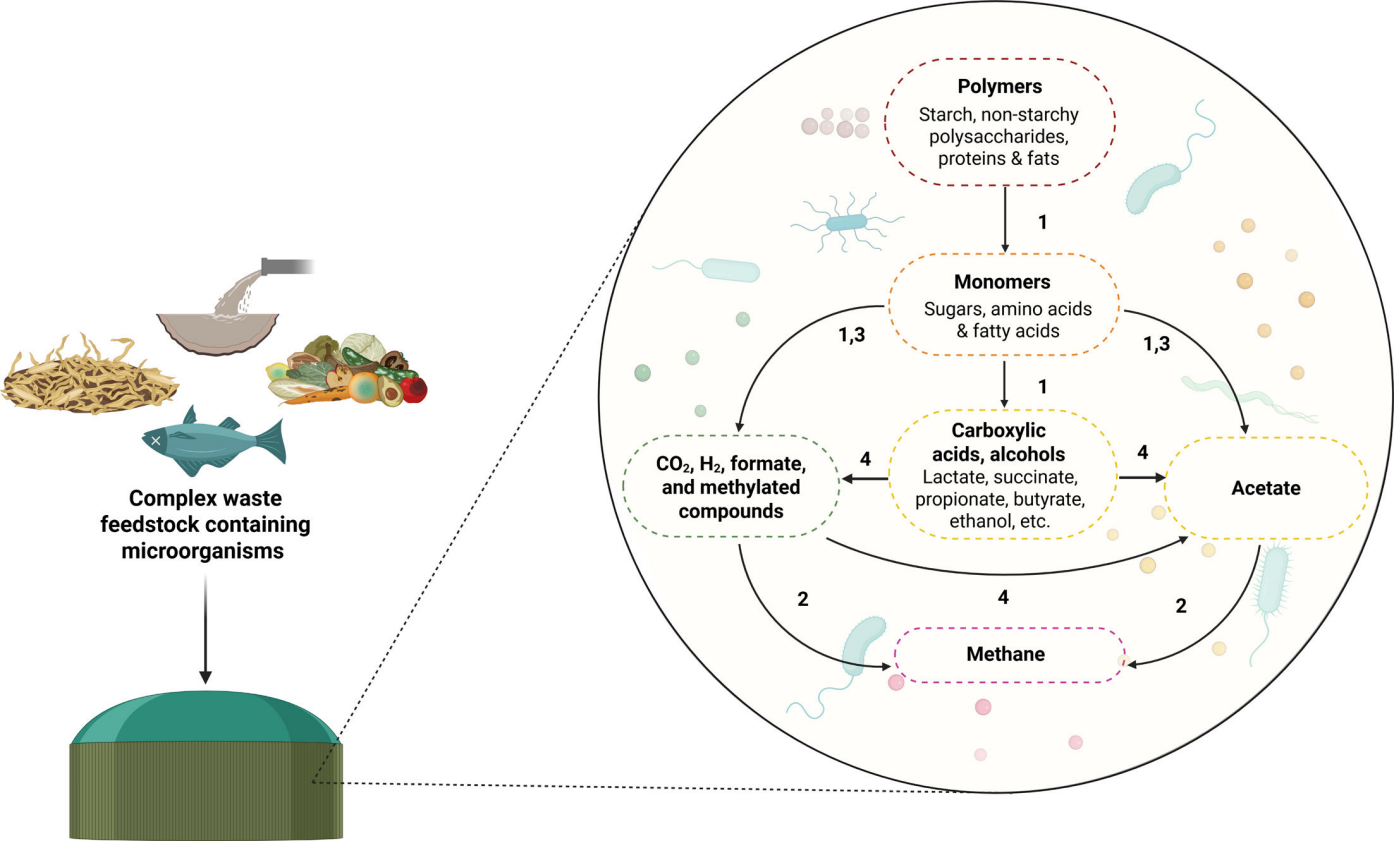
Illustration of simplified anaerobic food chain for waste digestion. Numbers denote different trophic groups: 1, primary fermenters; 2, methanogens; 3, secondary fermenters; 4, acetogens.

The different trophic groups of the anaerobic food chain have been characterized (Fig. 1) (23). In short, primary fermenters degrade complex biopolymers including carbohydrates, proteins, and lipids, into a wide range of fermentation products, including SCCAs (e.g., acetate, propionate, butyrate, formate, lactate, succinate, valerate), H_2_, and carbon dioxide (CO_2_). Some of the products, including H_2_, CO_2_, formate, and acetate, serve as direct substrates for methanogens to produce CH_4_ through methanogenesis. Some intermediate products are degraded by secondary fermenters to acetate, H_2_, and CO_2_, the process of which can only proceed when H_2_, formate, and acetate are used by methanogens to ensure the overall reaction remains thermodynamically favorable. Finally, acetogens connect the pool of one-carbon compounds and H_2_ with acetate through a process known as acetogenesis. Acetogenesis also includes the production of acetate by secondary fermenters. Overall, the digestion process is characterized by four sequential stages: hydrolysis, acidogenesis, acetogenesis, and methanogenesis carried out by different microbial guilds. Each stage produces metabolites that serve as substrates for subsequent reactions, enabling the stepwise conversion of complex organic matter to biogas. Descriptions of each stage are provided below, focusing on the microbial guilds involved (Table 1).

**TABLE 1:** Functional groups in anaerobic digestion

Biological functional groups (BFGs)	Trophic groups	Main substrates	Main metabolites	Main pathway	Representative genera	Reference
A: Acetate, propionate, succinate producers	Primary fermenters	Polysaccharides	Acetate, propionate, succinate, formate, H₂, CO₂	Mixed-acid fermentation	*Bacteroides,* *Enterobacter*	(24–26)
B: Non-butyrate-forming starch degraders	Primary fermenters	Starch, oligosaccharides, and sugars	Acetate, CO₂, H₂, ethanol	Acetate-ethanol-type fermentation	*Ruminococcus*	(27, 28)
C: Acetone, butanol, ethanol producers	Primary fermenters	Polysaccharides	Acetone, ethanol, butanol	Acetone-butanol-ethanol-type (ABE) fermentation	*Clostridium*	(29, 30)
D: Non-butyrate-forming fiber degraders	Primary fermenters	Cellulose, polysaccharides	Acetate, propionate, succinate, formate, H₂, CO₂	Mixed-acid fermentation	*Ruminococcus, Cellulosibacter*	(24)
E: Propionate producers 1	Primary fermenters	Polysaccharides, lactate	Propionate, acetate	Propionate-type fermentation (pyruvate reduction with lactate intermediate)	*Clostridium*	(31)
F: Propionate producers 2	Primary fermenters	Polysaccharides, lactate	Propionate, acetate	Propionate-type fermentation (transcarboxylase cycle)	*Propionibacterium, Bifidobacterium*	(27)
G: Lactate producers 1	Primary fermenters	Carbohydrates (mainly hexoses)	Lactate	Homolactate fermentation	*Lactobacillus*	(14, 29, 32)
H: Lactate producers 2	Primary fermenters	Carbohydrates (pentose and hexoses)	Acetate, lactate, ethanol, CO₂	Heterolactate fermentation	*Lactobacillus, Leuconostoc*	(27, 29)
I: Butyrate producers 1	Primary fermenters	Polysaccharides (including starch and cellulose)	Butyrate, acetate, H₂, CO₂	Direct conversion via butyrate kinase	*Clostridium*	(33, 34)
J: Butyrate producers 2	Primary fermenters	Acetate, lactate	Butyrate, acetate, H₂, CO₂	Indirect conversion via butyryl-CoA, acetate-CoA transferase, succinyl-CoA synthetase, and lactate dehydrogenase, respectively, and butyrate kinase	*Clostridium, Prevotella*	(33)
K: Amino acid degraders	Primary fermenters	Amino acids	Acetate, propionate, butyrate, valerate, CO₂, H₂	Stickland fermentation	*Clostridium, Aminobacterium*	(35–37)
L: Glycerol degraders	Primary fermenters	Glycerol	Succinate, pyruvate, propionate, 1,3-propanediol	Oxidative/reductive pathways	*Clostridium, Enterobacter, Klebsiella, Citrobacter*	(38–40)
M: Butyrate oxidizer 1	Secondary fermenters	H₂, acetate	Butyrate	β-oxidation	*Syntrophomonas*	(41)
N: Propionate oxidizer	Secondary fermenters	Propionate	H₂, acetate	Methylomalonyl-CoA pathway	*Syntrophobacter*	(41)
O: Acetate oxidizer	Secondary fermenters	Acetate	H₂, CO₂	Wood-Ljungdahl pathway	*Clostridium*	(23, 42)
P: Ethanol oxidizer	Secondary fermenters	Ethanol	Acetate	Ethanol oxidation	*Clostridium, Pelobacter, Desulfovibrio*	(43)
Q: Long-chain fatty acid degraders	Secondary fermenters	Long-chain fatty acids	Acetate, H₂	β-oxidation	*Treponema, Syntrophomonas, Syntrophus, Desulfomonilia*	(39, 44)
R: Lactate oxidizers	Secondary fermenters	Lactate	Acetate	Lactate oxidation	*Acetobacterium, Moorella*	(45, 46)
S: Acetogens	Acetogens	H₂, CO₂, sugars	Acetate	Wood-Ljungdahl pathway	*Acetobacterium*	(42, 47)
T: Hydrogenotrophic methanogens	Methanogens	H₂, CO₂, formate	CH₄	Hydrogenotrophic methanogenesis	*Methanococcus, Methanobacterium*	(41, 48)
U: Acetoclastic methanogens	Methanogens	Acetate	CH₄	Aceticlastic/acetotrophic methanogenesis	*Methanothrix, Methanosaeta, Methanosarcina*	(41, 49)
V: H₂-dependent methylotrophic methanogens	Methanogens	Methanol, methylamines, methylsulfides, H₂	CH₄	Methylotrophic methanogenesis	*Methanomassiliicoccus*	(29)
W: H₂-independent methylotrophic methanogens	Methanogens	Methanol, methylamines, methylsulfides	CH₄	Methylotrophic methanogenesis	*Methanosarcina*	(29)

#### Hydrolysis

Hydrolysis involves the breakdown of biopolymers such as cellulose, starch, proteins, and fats into monomers or oligomers of sugars, amino acids, and fatty acids. This step releases dissolved organic matter from the waste, such as cellular matters from activated sludge, and is primarily mediated by extracellular enzymes, including cellulases, proteases, and lipases, produced by primary fermenters (50). Hydrolysis is often regarded as the slowest and most limiting step in many anaerobic digestion processes, particularly for wastes with high lignocellulosic content (51, 52). Although some hydrolysis products can be converted directly to biogas, most require further transformation during the acidogenesis step (53). The microorganisms driving hydrolysis include bacteria from the phyla *Bacteroidetes, Firmicutes,* and *Proteobacteria,* as well as eukaryotic anaerobic fungi in genera *Neocallimastix*, *Piromyces,* and *Orpinomyces* (54).

#### Acidogenesis

Hydrolysis products are further metabolized into SCCAs, alcohols (e.g., ethanol and methanol), and minor intermediate products such as glycerol and acetone. The main SCCAs formed include acetate, propionate, and butyrate, typically in a ratio ranging from 75:15:10 to 40:40:20 (55). Byproducts such as CO_2_, H_2_, NH_3_, and trace metabolites are also generated. Acidogenesis progresses rapidly, potentially leading to acid accumulation, which can subsequently inhibit methanogenesis (53). The microorganisms involved in acidogenesis can be broadly classified into functional groups based on their metabolic pathways and primary fermentation products (56–58), as summarized in Table 1. Sugars are metabolized through various pathways, including acetate-ethanol fermentation (AET) (56–58), propionate-type fermentation (PTF) (27, 57, 59), mixed-acid fermentation (MAF) (29, 60), and lactate-type fermentation (LTF) (60). Amino acids undergo degradation through Stickland metabolism, where the oxidation of one amino acid is coupled with the reduction of another amino acid within the same cell (61–64). Amino acid oxidation can also be coupled to alternative reductive pathways, such as methanogenesis, either within a single organism or via interspecies electron transfer (23). Some amino acids can also be fermented individually (65, 66). Notably, amino acid cross-feeding plays a crucial role in shaping microbiome diversity, stability, and resilience (67–69). Lipids are hydrolyzed into glycerol and long-chain fatty acids. Glycerol can be reduced to 1,3-propanediol or oxidized into intermediates such as succinate and pyruvate, which enter glycolytic fermentation pathways (29, 38). Long-chain fatty acids are primarily degraded via β-oxidation, producing acetate and H_2_ in a syntrophy-dependent process (39, 40).

#### Acetogenesis

Acetogenesis involves the production of acetate either through CO_2_ reduction or the oxidation of organic acids. H_2_-utilizing acetogens are obligate anaerobes employing the acetyl-CoA pathway as their primary mechanism for synthesizing acetyl-CoA from CO_2_ (70–74). Organic acids such as propionate and butyrate, as well as alcohols like ethanol, are oxidized to acetate by H_2_-producing acetogens, with electrons transferred to protons or bicarbonate, forming H_2_ or formate, respectively (75).

Protons are critical terminal electron acceptors in anaerobic microbial communities, and H_2_ is produced via the oxidation of redox mediators such as NAD^+^/NADH, FAD/FADH_2_, and ferredoxin (oxidized/reduced). Certain acetogenic pathways rely on the consumption of metabolic end-products through syntrophic interactions with methanogens or sulfate reducers. For these processes to remain thermodynamically favorable, H_2_ partial pressure must be kept low to facilitate the regeneration of redox intermediates (23, 74, 76). Interspecies electron transfer plays a crucial role in this case, facilitating microbial cooperation. Electron carriers such as H_2_ and formate mediate these exchanges via redox couples H_2_/H^+^ (E°’ = −414 mV) and formate/CO_2_ (E°’ = −432 mV) (74). Additionally, direct electron transfer can occur through structures like cytochromes, nanowires, or conductive materials (50, 74, 77). Fermentative organisms are thus classified as obligate or facultative syntrophs based on their reliance on interspecies interactions. Facultative syntrophs can regenerate NAD^+^ by reducing intracellular metabolites, whereas obligate syntrophs depend on H_2_ scavengers to consume local H_2_ and sustain energetics. Obligate syntrophs can form cluster-like structures to enhance metabolic exchange efficiency (74).

#### Methanogenesis

CH_4_ is produced from acetate by acetoclastic methanogens or from H_2_ and CO_2_ by hydrogenotrophic methanogens. CH_4_ production can also occur via methylotrophic pathways, either H_2_ independent or H_2_ dependent. In the H_2_-independent pathway, methanogens oxidize one methyl group to CO_2_ to generate reducing equivalents needed to reduce another methyl group to CH_4_ (29, 78). In contrast, H_2_-dependent methylotrophs rely on externally supplied H_2_ as an electron donor for CH_4_ formation, as seen in *Methanomassiliicoccales* (78). The oxidation of propionate, butyrate, and lactate through syntrophic interactions is tightly associated with hydrogenotrophic methanogens (79).

The metabolic pathways and microorganisms involved in AD are highly diverse and influenced by environmental factors such as temperature, pH, H_2_ partial pressure (27, 80), hydraulic retention time, as well as co-factor and substrate availabilities (27, 81–83). These factors shape microbial composition and regulate competition and cooperation within the microbial community. For example, acetogens compete with hydrogenotrophic methanogens for H_2_ and CO_2_, with equilibrium dictated by pH and H_2_ partial pressure, while acetoclastic and hydrogenotrophic methanogens co-exist depending on H_2_ versus acetate flux, temperature, and pH (84, 85). Importantly, microorganisms also influence environmental conditions through metabolite production, creating a dynamic ecophysiological feedback loop that shapes community structure and function. The impact of operational parameters on AD performances has been previously reviewed (53, 55).

### Implications

Deciphering the AD microbiome and its ecological dynamics is challenging due to the complexity of the interspecies interactions, which fluctuate with environmental conditions both temporally and spatially in response to biotic and abiotic factors. The AD community is highly diverse and functionally redundant due to the heterogeneity of waste and the open nature of the system (86). In this context, a key question is whether the process can be controlled to consistently produce specific products (in addition to biogas) despite dynamic and variable microbial and environmental conditions. While SynComs can provide insights into AD ecology, their design raises questions about how to balance simplicity and functionality while maintaining the critical dynamics and adaptability inherent to natural ecosystems. It is crucial to evaluate whether insights from SynComs can be generalized to real-world communities. SynComs have the potential to serve as models to uncover universal principles of microbial interactions in AD, beyond the characteristics and complexities of individual ecosystems.

## DESIGNING AND MODELING AD SYNTHETIC COMMUNITIES

The design of SynComs involves constructing functionally representative consortia that can emulate key ecological and metabolic functions of interest from natural consortia while simplifying their complexity. By reducing the diversity to essential taxa, SynComs serve as effective models for studying microbial interactions and optimizing functional outcomes. This approach is based on the concept of core microbiomes, where a subset of microbial taxa supports critical functions in a given ecosystem (1).

An initial step is identifying and incorporating keystone taxa defined as highly connected organisms that, whether individually or within a guild, exert a disproportionate influence on community function and structure despite their abundance. These taxa play a unique and crucial role in maintaining community stability, exhibiting high connectivity. For example, syntrophic relationships are essential for maintaining stable AD processes (87). Importantly, the role of keystone taxa and microbial interactions within a community is highly context dependent, varying across environmental conditions and community composition (6, 88).

An effective design of AD SynComs should be tailored to specific research objectives (89), striking a balance between effective coarse-graining and simplification. For instance, mechanistic studies focused on specific degradation pathways may only require highly simplified communities. In contrast, investigating functional redundancy and its role in perturbation resistance demands more complex communities that resemble real-world AD microbiomes. SynComs designed to explore microbial interactions would likely fall somewhere in between. Moreover, SynComs design must consider key operational parameters, such as temperature, pH, substrate load, etc., which directly influence the identification of keystone species and their metabolic profiles and interactions. Studies examining the influence of these factors on both community-level functions and on pure and co-cultures could yield valuable insights for SynComs design.

### Identification of a core microbiome

One of the most widely used methods for defining the core microbiome is co-occurrence network analysis, which utilizes statistical associations to infer microbial interactions and identify keystone taxa (90, 91). For example, Narihiro et al. (92) applied co-occurrence analysis to a methanogenic community treating terephthalic acid wastewater and demonstrated that microbial pairs establish thermodynamically favorable relationships, optimizing energy efficiency. To determine the nature of the ecological interactions discovered by such analysis often necessitates validation through experimental approaches (6, 88). Structural equation modeling (SEM) is a promising tool for distinguishing causal relationships from correlations, bridging theoretical and experimental insights (88). Additionally, membership-based core analysis, which examines shared presence–absence patterns, and composition-based methods, which account for relative abundance, can be integrated with phylogenetic, persistence, and functional redundancy metrics to assess whether shared taxa fulfill similar ecological roles (1).

Identifying the core microbiome in AD is complex due to the vast diversity of low-abundance species. Large-scale initiatives like Microbial Database for Activated Sludge and Anaerobic Digesters (MiDAS) (41) have significantly contributed to bridging knowledge gaps on digester microbial compositions. This project synthesizes decades of data on essential microorganisms in wastewater treatment and AD systems, and can be used to guide the design of AD SynComs. Central to the findings is the identification of a core microbiome, consistently present across digesters regardless of geographic location. Species and genera were categorized into strict, general, and loose core groups based on their relative abundance across samples (41, 49, 93). The strict core includes species with a relative abundance > 0.1% in at least 80% of samples, the general core > 0.1% in >50% of samples, and the loose core > 0.1% in >20% of samples. For example, at the genus level, organisms identified as the strict core microbiome include 11 known methanogenic genera (*Ca. Methanofastidiosum, Ca. Methanoplasma, Methanobacterium, Methanobrevibacter, Methanoculleus, Methanolinea, Methanomassiliicoccus, Methanosarcina, Methanospirillum, Methanothermobacter,* and *Methanothrix*) and several syntrophic bacteria including the genera *Smithella, Syntrophomonas,* and *Syntrophorhabdus*. At the species level, the strict core contained methanogens *Methanobrevibacter smithii* and *Methanothermobacter midas_s_3958*, as well as the syntrophic bacteria *Syntrophomonas midas_s_90707* (41). This list combines strict core genera from digesters treating different substrates (food waste, industrial waste, manure, wastewater sludge) and operating temperatures (mesophilic versus thermophilic). The analysis concluded that while most core genera were uniquely associated with specific substrates and temperatures, a significant number were shared across substrates. In contrast, very few core species were shared across different substrates. Treu et al. (94) also observed a core microbiome that persisted across varying operational conditions, comprising key taxa such as *Methanoculleus, Methanothermobacter, Syntrophomonas,* and members of *Proteobacteria*. Their study highlighted that archaeal populations exhibited greater stability, whereas bacterial communities demonstrated higher diversity, likely due to their greater functional variability. Similar patterns have been observed in other studies (95, 96). Dueholm et al. (41) also identified conditionally rare or abundant taxa (CRATs), which are not consistently present but can dominate in response to environmental changes, significantly contributing to community function. Despite representing only a fraction of microbial diversity, CRATs enhance system resilience and recovery, highlighting the ecological flexibility of AD systems (32, 97).

### Core microbiome based on metabolic functions

Despite the high taxonomic diversity in AD communities, many taxa can be grouped into functional guilds based on shared metabolic traits, highlighting the functional redundancy of the system. This redundancy ensures that critical functions persist even if certain taxa are lost (5, 6). Grouping microbes into guilds reduces system complexity by clustering organisms with similar ecological roles, offering a biologically relevant framework for studying microbial interactions (86). In AD and related ecosystems, such as animal digestive systems, microbial communities have been classified into functional guilds according to their trophic roles and fermentation pathways (24, 98, 99). For instance, Kettle et al. (99) categorized 10 biological functional groups (BFGs) in the human colonic community based on key metabolic and physiological characteristics, including substrate preferences, fermentation byproducts, and optimal pH ranges, and used these to build a mathematical model predicting community metabolic products.

**Fig 2:**
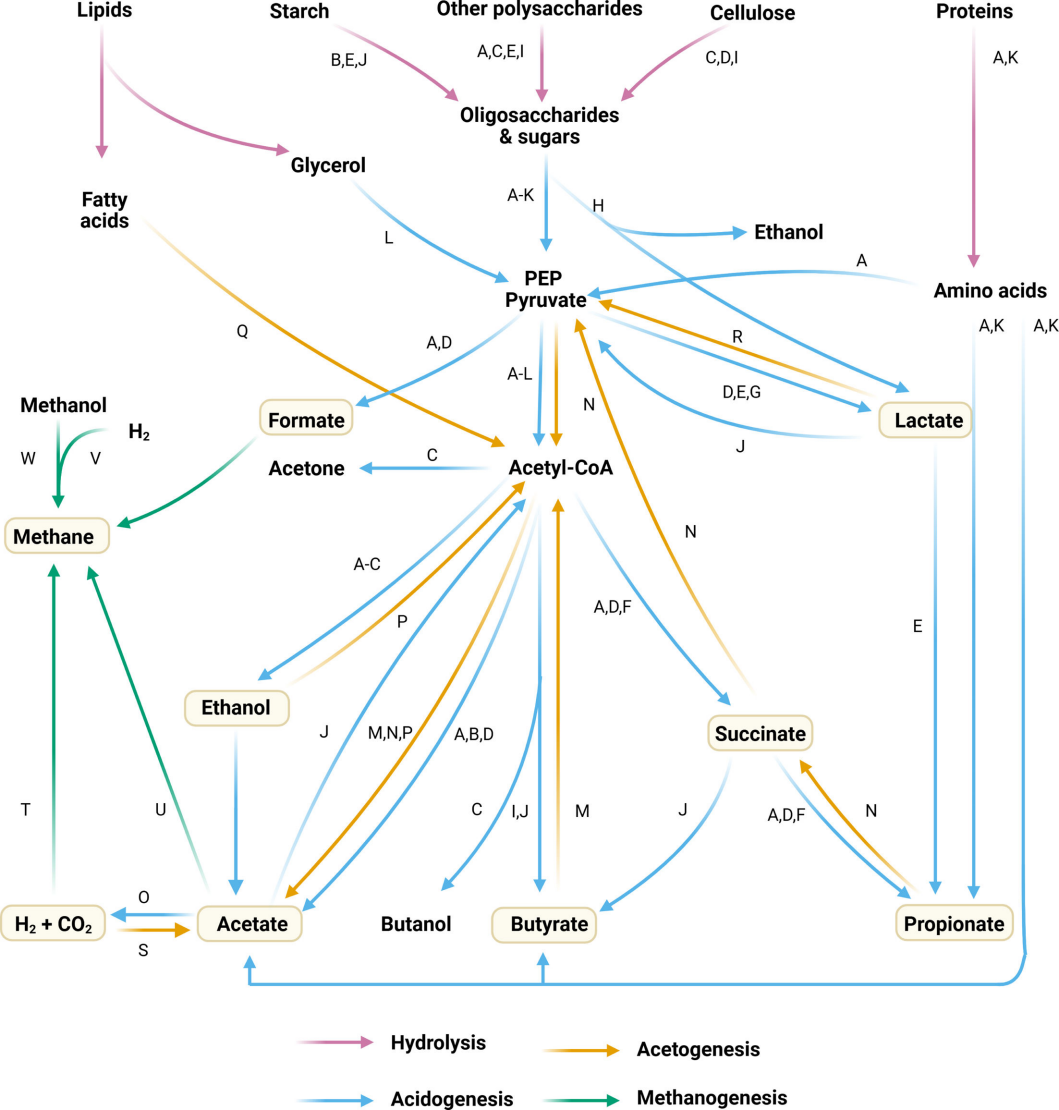
Simplified representation of central metabolic pathways in anaerobic digestion. Functional groups are denoted by letters, with their correspondence provided in Table 1. Acetyl-CoA, acetyl coenzyme A; PEP pyruvate, phosphoenolpyruvate.

In Table 1, we propose a framework for classifying the AD community into BFGs, inspired by previous classifications (33, 99). Representative genera were selected based on their documented presence in ADs, with some also commonly found in the human gut or rumen. A simplified overview of the metabolic interactions between BFGs is presented in Fig. 2. The construction of Table 1 emphasizes the acidogenesis step, highlighting the dynamic and cooperative microbial interactions that underpin the digestion process. Stickland metabolism of amino acids was considered, while other pathways were not considered, and sulfate presence is hypothesized as a potential factor influencing specific metabolic pathways, as described by Zhang et al. (35). Eukaryotic species are excluded.

It is worth noting that certain microbial genera exhibit great versatility, exemplified by *Clostridium*, which includes species across several BFGs. Furthermore, some microbes can conditionally shift between BFGs, as their metabolic activity depends on environmental conditions and interactions with other members. For instance, some acetogens reduce CO_2_ with H_2_ to produce acetate, but in the presence of methanogens, they can shift to converting sugars and lactate, yielding acetate and H₂ (100–104).

### Experimental approach

Both top-down and bottom-up approaches are valuable strategies for working with SynComs (105). Top-down methods, such as community enrichment, aim to simplify native communities by enriching or selecting for certain interactions or functions while preserving ecological relevance by leveraging existing microbiome functionalities. These methods can provide insights into how microbial communities respond to abiotic stresses and environmental fluctuations, helping to uncover mechanisms of acclimatation and stability. Several studies have demonstrated the effectiveness of top-down strategies in enhancing the production of metabolites from waste streams, including biogas, carboxylic acids, and PHAs (105, 106). For example, Gilmore et al. (107) enriched a minimal lignocellulose-degrading community comprising four microbial members from equine feces that produces biogas 0.75- to 1.9-fold more at 1.4–2.1 times the rate compared to a monoculture of fungi. The community is more stable than a bottom-up assembled community composed of different strains with similar functions, highlighting the advantage of leveraging naturally occurring microbial communities. While the top-down approach is effective in narrowing down essential taxa or metabolic pathways within complex systems, it offers limited control over specific community members if the enriched taxa are not isolated and well characterized (108). Depending on the selection strategy, simplified communities may exclude rare but functionally important taxa, such as those contributing to system robustness to disturbance.

In contrast, bottom-up approaches enable systematic drop-out and drop-in experiments, as well as pairwise interaction studies, allowing for the investigation of the specific roles and functional redundancy of individual species. These strategies rely on prior knowledge of the physiology and metabolism of community members, as well as potential interactions between them (105). For example, Wang et al. (109) co-cultured key H_2_-scavenging microbes: *Blautia hydrogenotrophica*, *Desulfovibrio piger*, and *Methanobrevibacter smithii*, thereby revealing that their relationships are not simply competitive but can coexist or even stimulate, which are condition dependent. This study underscored the utility and effectiveness of such methods for dissecting interaction mechanisms (68). However, the results and their interpretation may be constrained by the artificial selection process. Strain selection can be guided by various strategies (110), including enrichment communities and trophic-based selection. The latter focuses on assembling species that collectively capture key metabolic pathways and functional traits of the target microbiome, fostering metabolic dependencies that promote stable community formation (111). Several studies have demonstrated the potential of bottom-up assembled SynComs for applications in human health (112–114), typically involving detailed strain characterization followed by co-culture experiments. Pairwise interaction studies map direct interactions between species, shedding light on cooperative and competitive dynamics. The ability of pairwise analyses to capture higher-order interactions has been discussed, with findings showing that it depends on factors such as the number of species in the assemblage (115, 116). Lyu et al. (105) reviewed studies utilizing both bottom-up and top-down approaches, providing a more detailed discussion in their work.

### Modeling of microbial interactions

Models of microbial interactions not only facilitate the rational design of SynComs by providing insights into microbial relationships, resource utilization, and metabolic potential, but also enable the prediction of metabolite accumulation, whether beneficial or inhibitory, thereby supporting precise control over community behavior in various applications. These models are generally classified into two categories: those with defined interactions and those with resource-mediated interactions (108).

**Generalized Lotka-Volterra (gLV) models** belong to the first category. These models describe direct microbial interactions, categorized into six archetypes (108, 117), and assume interaction coefficients remain constant over time and across environmental changes. Using differential equations, they track species abundance based on growth rates, self-limitation, and pairwise interactions. While higher-order interactions can be incorporated, gLV models primarily rely on pairwise data to assess system sensitivity to perturbations. These models do not predict how nutrient diversity or availability influences community richness over time (108, 115). As species diversity increases, parameterization becomes labor-intensive. High-throughput tools like kChip streamline this process by simultaneously testing hundreds of pairwise interactions (105, 118), significantly reducing experimental workload and making gLV models more feasible for studying diverse systems. Despite their utility, gLV models are underutilized in AD research due to the complexity of their parameterization, which requires further refinement for effective application in AD systems (119).

**Consumer-resource models** emphasize metabolite exchange within microbial communities (120), incorporating spatial structure where microbial proximity affects metabolite diffusion and direct transfer. Parameterization relies on detailed substrate consumption and excretion data from growth characterization and metabolomics (108). Despite the need for such extensive data, consumer-resource models are particularly effective for studying metabolic cross-feeding and trophic interactions, making them a powerful tool for optimizing nutrient use.

**Genome-scale metabolic network models (GEMs**) can optimize metabolic fluxes, division of labor, and ecological niche occupation in microbial communities (121). Representing cellular biochemistry as a stoichiometric matrix, GEMs are generated from annotated genomes (122) or manually curated for greater accuracy. Once a GEM is constructed, it can be analyzed using flux balance analysis (FBA), a constraint-based optimization method that predicts metabolic fluxes under a steady-state assumption (11). FBA calculates flux by optimizing a target function, typically biomass production (11, 121). While computationally efficient and scalable, FBA is static and does not capture dynamic microbial interactions or environmental fluctuations. To overcome this, dynamic FBA variants, such as SteadyCom, incorporate additional constraints for multi-species systems to more accurately model balanced growth (11). To further address FBA limitations, innovative tools such as ASTHERISC have been developed, integrating thermodynamic constraints to enhance metabolite production by distributing metabolic pathways across multiple microbial strains, and thus optimizing multi-strain configurations for targeted outcomes. However, they still lack the ability to incorporate considerations such as ensuring long-term community stability. Combining GEMs with FBA enables detailed metabolic and interaction analysis, and can facilitate community design. For example, Basile et al. (10) combined large-scale GEMs with pairwise FBAs to predict that symbiotic interactions are more prevalent than antagonistic ones and that amino acid exchange may be prevalent in AD communities.

Significant efforts have been dedicated to the development of AD models aimed at predicting system performance, enhancing biogas production, and improving process stability by simulating microbial interactions and physicochemical transformations (123). They offer insights into metabolic interactions within microbial consortia. Existing models range from empirical approaches that rely on statistical correlations to kinetic models that describe microbial growth and substrate degradation (123–126). Among them, the Anaerobic Digestion Model 1 (ADM1) is the most widely used structured model, offering a comprehensive representation of AD processes (127, 128). Despite their utility, AD models face challenges such as high data demands, parameter uncertainty, and computational complexity, which limit their practical application (126). The diversity of AD processes also impacts model relevance, necessitating adaptations for different operational conditions. An effective AD model must balance between accuracy, robustness, simplicity, flexibility, reasonable data requirements, and computational efficiency (124), and great efforts have been directed toward improving existing models for AD, notably with the Anaerobic Digestion System Model (ADSM) to provide a more reliable reference point for AD simulations (129).

### Practical considerations of working with AD SynComs

**Cultivability** is often a limiting factor, as the majority of AD microorganisms remain unisolated, likely due to an incomplete understanding of their growth requirements and potential dependence on obligatory partnerships. Given their natural habitats in waste environments, many species likely rely on metabolite exchange for sustained growth. Specialized cultivation methods, such as gradient culture systems, cocultures, and microfluidic techniques, may help address this challenge (81, 130). Metagenome-assembled genomes (MAGs) could also offer insights into their auxotrophic needs and enable the development of more precise isolation techniques. Notably, a considerable overlap exists between the species found in digesters and those in the gut, suggesting the successful methods in gut microbiome research may be adapted for this context.

Moreover, working with **genetically characterized organisms** offers advantages, as genome analysis provides insights into the strains’ metabolic capabilities. This knowledge is crucial for predicting interactions and engineering functional outputs, such as optimizing cross-feeding interactions or minimizing competitive exclusions (10, 98, 115). Additionally, utilizing well-annotated genomes facilitates the integration of multi-omics approaches and modeling of community-level interactions and metabolic fluxes (11).

The **growth rates** of selected organisms should ideally fall within a close range to maintain balanced community dynamics. However, achieving this can be challenging due to the inherent limitations of certain metabolic pathways. Therefore, the experimental design should carefully determine parameters such as transfer time and dilution rate in bioreactors to ensure the stability of the intended species composition. Doubling times vary widely among anaerobic microorganisms, with fermentative species like *Anaeromicropila populeti* exhibiting a doubling time of approximately 2.5 hours in batch cultures when grown on glucose (131), whereas acetoclastic and hydrogenotrophic methanogens display doubling times of 10 hours and 2 hours and longer, respectively (9, 132). Syntrophic bacteria, reliant on interspecies interactions for growth, grow even slower. For example, *Syntrophomonas sapovorans* and *Syntrophomonas wolfei* subsp*. wolfei* double approximately every 40 and 90 hours, respectively, when utilizing butyrate under optimal conditions (133). To mitigate competition and ensure that slower-growing organisms are not outdiluted, species can be introduced at staggered intervals, allowing the community to synchronize its growth dynamics. It is also crucial to recognize that growth rates are highly dependent on environmental parameters such as nutrient availability (134).

Designing a **cultivation medium** that supports all community members is critical. The medium should account for the metabolic requirements of each organism while preserving metabolic interactions, such as cross-feeding, and ensure a stable and functional community composition (99, 135). The choice between a complex versus a defined medium depends on the priority of either closely representing the AD environment or probing a specific targeted functionality in a highly controlled manner. Using low-nutrient media has been found to help maintain the functional diversity of a community (136). The concentration of nutrients should be carefully considered to ensure consortium stability, including the selection of an appropriate pH buffering system to account for the production of SCCAs. A great number of digester isolates require complex components such as meat extract to grow, which may also serve as an energy substrate for the designed community and introduce uncertainty in metabolic analysis. In designing a butyrate-producing community, Clark et al. (4) demonstrated that undefined components such as mixes of amino acids can be systematically replaced with defined alternatives through an iterative process. Moreover, it is important to note that certain organisms have specific growth requirements. For example, methanogens depend on reduced sulfur sources and typically cannot assimilate sulfate, with the reported exception of *Methanothermococcus thermolithotrophicus* (136, 137). However, reduced sulfur sources like hydrogen sulfide can be toxic to certain species (138). Nitrogen metabolism in AD often relies on cross-feeding from, for example, amino acid degradation where ammonia is produced through deamination reactions. However, ammonia accumulation can inhibit microbial activity, particularly methanogenesis.

**Cultivating anaerobic species** presents inherent challenges due to the labor-intensive nature of working with sealed bottles, inside an anaerobic glovebox, or reactors. Fine-tuning experimental parameters and conducting high-throughput characterization can be difficult, especially when gas control or measurements are needed. Commercial microplate readers designed for microbial growth assays can be placed inside an anaerobic glovebox. A sulfide removal column is important to prevent the accumulation of reduced sulfur compounds produced during anaerobic degradation in the chambers, particularly when using rich media (131), from damaging electronic components. Traditional gloveboxes remove O_2_ by maintaining a low concentration of H_2_ (<5%) through a palladium-catalyzed reaction. Because H_2_ is a key intermediate metabolite in digestion, its presence may influence microbial cultivation and metabolism. Some newer glovebox designs eliminate H_2_ from the main chamber by circulating gas into a side compartment for O_2_ removal, thereby mitigating its impact on microbial communities.

**Cryopreservation** is crucial for the long-term storage and reproducibility of SynComs across laboratories and over time, facilitating collaborative and comparative research (135). Protocols must preserve microbial viability and community structure, ensuring consistent recovery of individual species or consortia. Some fermenters are O_2_ tolerant and can be revived from cryostocks prepared under aerobic conditions. However, others are more sensitive and require preparation under N_2_ flushing or within an anaerobic glovebox.

### Validation of SynComs and relevance of findings

Experimental validation is essential to ensure that SynComs accurately model target ecosystems and fulfill their intended functions. Validation typically involves (i) confirming community composition over time through sequencing-based methods (135, 139–141), (ii) quantifying metabolic fluxes and key intermediates via metabolomics to assess functionality (4, 111), and (iii) testing resilience through perturbation experiments, such as pH shifts, substrate fluctuations, species drop-in/drop-out (98, 142). The choice of experimental platform should align with the research objectives—for example, continuous bioreactors are suitable for studying stable processes, while batch systems are better suited to capturing transient interactions.

Furthermore, the relevance of the findings derived from simplified communities should be evaluated with more complex or real-world systems. Experimental frameworks for validation should be tailored to the specific hypotheses driving SynComs design and employ strategies that align with the original design objectives. For instance, validation of degradation pathways identified through SynComs can be achieved by challenging the community with complex, real-world substrates to assess ecological relevance. To determine whether identified interactions or functions persist in natural settings, one can conduct perturbation experiments (143), analyze changes in gene expression levels of relevant pathways (144, 145), or use stable isotope labeling to trace metabolite exchanges (146–148) within real-world AD communities.

## APPLICATION OF SynCcoms FOR ANAEROBIC DIGESTION

As shown in Table 2, the application of SynComs to study AD processes and microbial ecology is still in the early stages. Although the number of studies remains limited, progress has been made in enhancing the degradation of recalcitrant, lignocellulosic substrates. These studies have focused on boosting biogas production (107, 149–152) or carboxylic acid production (153–155), using two main strategies: enrichment from complex consortia to achieve specific functions or rational design based on metabolic capabilities.

**TABLE 2:** Studies of SynComs in anaerobic digestion processes

Reference	Organisms	Objective	Design strategy and approach
(149)	*Bacillus subtilis, Acinetobacter johnsonii, Trichoderma viride, Aspergillus niger*	Enzyme production for lignocellulose degradation	Bottom-up, functional selection
(150)	*Bacillus* sp*., Delftia* sp*., Pseudomonas* sp*., Lysinibacillus fusiform, Arthrobacter nicotianae, Paenibacillus ehimensis, Aspergillus* sp*., Trichoderma* sp.	Rice straw degradation for biogas production	-
(109)	*Ruminiclostridium cellulolyticum, Methanospirillum hungatei, Methanosaeta concilii, Desulfovibrio vulgaris*	Cellulose degradation and the cooperation of methanogens, acetogen, and sulfate-reducing bacteria for biogas production	Bottom-up, functional selection
(156)	Combinations of hydrogen-producing bacteria (*Clostridium butyricum* and *Clostridium beijerinckii*) and lactic acid bacteria (*Lactobacillus plantarum* and *Lactobacillus pentosus*)	Enhancing biogas production from food waste via bioaugmentation	Bottom-up
(157)	Mix of hydrogenotrophic methanogen species containing *Methanobacterium*	Methane production	Top-down
(158)	*Methanobrevibacter thaueri, Pecoramyces ruminantium*	Biogas production from biomass rich in lignocellulose	Top-down
(159)	Rumen cultures containing *Methanosarcina*	Bioaugmentation of digestion of food and vegetable wastes under feed composition fluctuations	-
(151)	Mixed culture containing *Bacteroidetes, Proteobacteria, Firmicutes, Spirochaetes, Actinobacteria*	Enhancing lignocellulosic substrate degradation for biogas production	Top-down
(160)	Cellulolytic culture mixture containing *Clostridium*	Degradation of cellulolytic waste	-
(107)	*Methanobacterium bryantii, Neocallimastix californiae, Anaeromyces robustus*	Lignocellulose degradation and biogas production	Top-down, bottom-up
(161)	Anaerobic ruminal fungi and hydrogen-producing fermenting bacteria	Bioaugmentation strategy for biogas production from wheat straw and mushroom spent straw	-
(162)	Enrichment culture containing *Methanothrix, Methanoculleus, Methanobacterium, Methanospirillum, Methanosarcina, Methanosphaerula, Methanomassiliicoccus, Methanosphaera, Methanoregula, Syntrophobacter*	Enhancing digestion efficiency for high C/N ratio feedstock	Top-down
(163)	Enriched cultures containing *Thermoanaerobacterium thermosaccharolyticum, Caldanaerobacter subterraneus, Thermoanaerobacter pseudethanolicus, Clostridium cellulolyticum*	Enhancement of methane production from cellulose and corn stover via bioaugmentation	Top-down
(164)	Moderately aerated, propionate-utilizing, mixed methanogenic enrichment culture	Improvement of methane production	Top-down
(165)	*Saccharomyces cerevisiae* sp*., Coccidioides immitis* sp*., Hansenula anomala* sp*., Bacillus licheniformis* sp*., Pseudomonas* sp*., Bacillus subtilis* sp*., Pleurotus florida* sp.	Bioaugmentation strategies for corn straw biogas production	Bottom-up
(166)	Rumen cultures containing *Rikenellaceae, Clostridiaceae, Porphyromonadaceae, Bacteroidaceae, Ruminococcaceae*	Enhancing biogas production from cow manure via bioaugmentation	Top-down
(167)	*Pseudomonas lundensis* DSM6252*, Methanoculleus bourgensis* MS2	Bioaugmentation of stressed batch reactors	Top-down
(168)	*Methanobrevibacter* spp*., Methanosarcina* spp.	Mitigation of ammonia inhibition through bioaugmentation	Bottom-up
(169)	Different consortia containing *Sporosarcina psychrophila, Comamonas aquatica, Shewanella baltica, Pseudomonas* sp. C27, *Brevefilum fermentans*	Bioaugmentation of anaerobic digesters with an enriched lignin-degrading consortium	Top-down
(170)	Bioaugmentation-enriched bacteria (*Bacteroidota, Synergistota*), anaerobic fungi (*Neocallimastigomycota*), and methanogens (*Methanosaeta, Methanothermobacter*)	Bioaugmentation of lignocellulosic AD methane production	Top-down

Mechanistic investigations, often conducted with a small number of microbial members, have yielded valuable insights into microbial interactions and their effects on digestion efficiency, as illustrated in the example discussed above (109). Several studies have also explored the use of SynComs as bioaugmentation agents to enhance specific functions or improve resistance to stress (25, 26, 28, 147–155, 171–176). While this strategy showcased the potential of practical applications of SynComs, its success has been moderate, possibly due to the limited understanding of the complex and dynamic nature of AD communities. The selection of microbial strains should consider their functional roles, adaptability, and sustained activity within the AD community (171, 172). Moreover, large-scale implementation of bioaugmentation strategies must consider the optimization of inoculum dosage, repetition, and timing, as studies have shown that while initial dosages correlate positively with reactor performance, this effect may diminish in subsequent applications, highlighting the need for tailored approaches to maintain long-term stability (173).

## CHALLENGES AND FUTURE WORK

SynComs show promise for both mechanistic studies and practical applications in enhancing AD processes. Their greatest strength likely lies in mechanistic studies that dissect microbial interactions. It is crucial to validate the conditional relevance of the findings in complex systems using real-world waste streams and native AD communities. The direct application of SynComs to industrial settings remains challenging. Bioaugmentation is likely a viable strategy; however, ensuring the long-term stability of designed communities or introduced species within AD communities is difficult and must be carefully evaluated. While sterilized waste streams may allow tighter microbial control, such approaches can be expensive and impractical at scale. Moreover, biosafety and ecological risks of introduced strains should be considered, given that waste treatment facilities are recognized hotspots for the emergence and spread of mobile antibiotic resistance genes (174).

SynComs are unlikely to capture the full complexity of real-world AD microbiomes. Balancing functional specificity, experimental feasibility, and resilience to perturbations is challenging. Resilience often relies on functional redundancy and low-abundance taxa, which are typically excluded from SynComs design. Therefore, it is essential to align research or application goals with SynComs design, as these objectives should inform the appropriate level of complexity. Improving SynComs design requires more knowledge gained from isolating and characterizing the ecophysiology of AD microbes, which can be facilitated by high-throughput cultivation and collaborative data annotation. Additionally, machine learning holds promise for modeling microbial interactions and incorporating environmental dependencies.

Despite the challenges, SynComs present significant potential to illuminate key microbial interactions—such as hydrogen, formate, and amino acid exchanges—that underpin AD function. Notably, SynComs development has progressed further in analogous systems like the gut microbiome, with diverse design strategies reviewed by Clavel et al. (175) and Van Leeuwen et al. (176). These insights can guide AD research moving forward. SynComs can be tailored for specific goals such as biogas production, medium-chain carboxylic acid synthesis, or lignocellulose degradation, contributing to system optimization and technological innovation. Collectively, these advancements support broader efforts in resource recovery and the circular bioeconomy.
